# Heart rate variability-derived features based on deep neural network for distinguishing different anaesthesia states

**DOI:** 10.1186/s12871-021-01285-x

**Published:** 2021-03-02

**Authors:** Jian Zhan, Zhuo-xi Wu, Zhen-xin Duan, Gui-ying Yang, Zhi-yong Du, Xiao-hang Bao, Hong Li

**Affiliations:** 1 Department of Anaesthesiology, The Second Affiliated Hospital of Army Medical University, Chongqing, 400037 China; 2 Department of Anaesthesiology, The Affiliated Hospital of Southwest Medical University, Luzhou, 646000 Sichuan China

**Keywords:** Depth of anaesthesia, Heart rate variability, Deep neural network, Discrete wavelet transform

## Abstract

**Background:**

Estimating the depth of anaesthesia (DoA) is critical in modern anaesthetic practice. Multiple DoA monitors based on electroencephalograms (EEGs) have been widely used for DoA monitoring; however, these monitors may be inaccurate under certain conditions. In this work, we hypothesize that heart rate variability (HRV)-derived features based on a deep neural network can distinguish different anaesthesia states, providing a secondary tool for DoA assessment.

**Methods:**

A novel method of distinguishing different anaesthesia states was developed based on four HRV-derived features in the time and frequency domain combined with a deep neural network. Four features were extracted from an electrocardiogram, including the HRV high-frequency power, low-frequency power, high-to-low-frequency power ratio, and sample entropy. Next, these features were used as inputs for the deep neural network, which utilized the expert assessment of consciousness level as the reference output. Finally, the deep neural network was compared with the logistic regression, support vector machine, and decision tree models. The datasets of 23 anaesthesia patients were used to assess the proposed method.

**Results:**

The accuracies of the four models, in distinguishing the anaesthesia states, were 86.2% (logistic regression), 87.5% (support vector machine), 87.2% (decision tree), and 90.1% (deep neural network). The accuracy of deep neural network was higher than those of the logistic regression (*p* < 0.05), support vector machine (*p* < 0.05), and decision tree (*p* < 0.05) approaches. Our method outperformed the logistic regression, support vector machine, and decision tree methods.

**Conclusions:**

The incorporation of four HRV-derived features in the time and frequency domain and a deep neural network could accurately distinguish between different anaesthesia states; however, this study is a pilot feasibility study. The proposed method—with other evaluation methods, such as EEG—is expected to assist anaesthesiologists in the accurate evaluation of the DoA.

**Supplementary Information:**

The online version contains supplementary material available at 10.1186/s12871-021-01285-x.

## Background

Both the central nervous and autonomic systems are related to the depth of anaesthesia (DoA) [1]. A DoA that is too shallow increases the risk of intraoperative awareness [2], and a DoA that is too deep can cause delayed recovery [3], cognitive dysfunction, and may increase the risk of death [4]. Therefore, accurate DoA monitoring is crucial to reducing the complications associated with overdose or insufficiency of anaesthetics and guarantying the safety and quality of anaesthesia.

However, the mechanisms of action of general anaesthetics are still not completely understood [5, 6], and there is currently no ‘gold standard’ for evaluating DoA [7]. DoA monitors based on electroencephalograms (EEGs) signals, such as bispectral index (BIS), Narcotrend, and entropy, have been widely used during surgery [8–10]. However, EEG signals only show the functions of the central nervous system and the indices based on these signals are not sufficiently accurate to assess DoA under certain conditions [11–15]. Therefore, it is essential to seek new methods of DoA monitoring to overcome the drawbacks of mainstream methods based on EEG signals [16] and improve the DoA monitoring accuracy. Electrocardiograms (ECGs) are internationally used in standard monitoring during general anaesthesia [17]. In addition, the heart rate variability (HRV) derived from an ECG is regulated by the central nervous and autonomic systems, and closely related to the DoA during surgery [18–20]. Therefore, HRV may be used as an important supplementary method of EEG monitoring in terms of DoA evaluation [21, 22].

Owing to the strong nonlinear characteristics of the EEG and ECG, nonlinear analysis methods may be used in studies of anaesthesia [23, 24]. Sample entropy (SampEn) is a typical nonlinear analysis method that was developed to study the time-domain features of HRV [25, 26] and provide an improved assessment of DoA during surgery [27, 28]. In addition, three frequency domain features of HRV, including the high-frequency power (HF), low-frequency power (LF), and ratio of high-to-low-frequency power (HF/LF), are related to the autonomic nervous system and have been implemented in anaesthesia research [29, 30].

Recently, several machine learning algorithms, including logistic regression [31], support vector machine [32], decision tree [33], artificial neural network [34], and deep neural network [35], have been utilized to assess DoA based on different time- and frequency-domain features of an EEG signal. These results indicate that it is necessary to combine multiple time and frequency domain features to improve DoA assessment methods. Moreover, to our knowledge, there are currently few studies combining HRV-derived features with machine learning algorithms to identify different anaesthesia states. Thus, we propose the hypothesis that multiple time and frequency features of HRV based on a deep neural network could be used to distinguish different anaesthesia states and provide a key supplementary method for EEG monitoring in the assessment of DoA.

## Methods

This study protocol was approved by the Institutional Ethics Committee of the Second Affiliated Hospital of the Army Medical University on March 25, 2020 (Chongqing, China, approval number: 2020–078-01). Patients were recruited from March 27, 2020 to April 29, 2020. Written informed consent was obtained from each patient. Twenty-three American Society of Anaesthesiology (ASA) physical status I or II adult patients, aged from 20 to 70 years old, scheduled to undergo elective laparoscopic abdominal surgery were recruited. Exclusion criteria included patients with neurological and cardiovascular diseases or a known allergy history of anaesthetics.

All patients underwent preoperative fasting for at least 8 h. The placement of the chest electrodes was the same for all participants. The five-leads were located at five different positions on the chest. The upper left position was at the junction of the midclavicular line on the left edge of the sternum and the first intercostal space. The lower left position was at the junction of the left midline of the clavicle and the level of the xiphoid process. The upper right position was at the junction between the midclavicular line on the right edge of the sternum and the first intercostal space. The lower right position was at the horizontal junction of the right clavicle midline and the xiphoid process, and the middle position was at the fourth intercostal space on the left edge of the sternum. After the electrodes were placed on the patient chest wall, anaesthesia was usually induced with intravenous midazolam, propofol, sufentanil, and cisatracurium. Loss of consciousness (LOC) was defined as no response to verbal commands and was tested every thirty seconds during anaesthesia induction [36]. Sevoflurane together with propofol and remifentanil were used to maintain anaesthesia. Recovery of consciousness (ROC) was defined as opening eyes following commands and was tested every one minute during anaesthesia recovery [36]. Table 1 summarises this information. Physiological signals (such as ECG, BP, HR, and SpO_2_) were measured to guarantee the safety of the patients under different anaesthesia states. The attending anaesthetist adjusted the DoA accordingly based on the observed signals and personal experience. From the various monitoring feedback information observed, attending anaesthetists need to analyse, synthesize, and judge the vital function indicators of patients according to their own experience and to make timely adjustments and interventions as needed to keep the vital signs as normal or close to the normal physiological state as possible, to adjust the DoA and maintain it at an appropriate level.

**Table 1:** Patients demographics and clinical characteristics

Parameters	means (SD)
Age (year)	50.2 (7.0)
Height (cm)	160.6 (6.9)
Weight (kg)	61.1 (9.4)
BMI (kg m^− 2^)	23.7 (3.2)
Duration of surgery (min)	132.9 (48.4)
Anaesthetic management	/
Midazolam induction (mg)	3.0 (0.8)
Propofol induction (mg)	62.0 (10.3)
Sufentanil induction (μg)	20.2 (2.7)
Cis-atracurium (mg)	13.1 (1.9)
Maintenance drugs infusion rate	/
Sevoflurane maintenance (Vol%)	1.7 (0.4)
Propofol maintenance (mg•kg^−1^ h^−1^)	2.1 (0.3)
Remifentanil (μg•kg^−1^ h^− 1^)	0.1 (0.04)
Additional drugs administrated when approaching the end of surgery	/
Sufentanil (μg)	7.1 (3.2)
Atropine (mg)	0.3 (0.1)
Neostigmine (mg)	0.7 (0.2)

Values are means (SD). *BMI* body mass index

In this study, ECG signals were recorded from twenty-three adult patients under general anaesthesia. The signals were recorded using a Philips MP60 monitor (Intellivue; Philips, Foster City, CA, USA). The operation time was 1—3 h. Raw ECG data were sampled at a 500-Hz sampling frequency.

### Expert assessment of consciousness level

The expert assessment of consciousness level (EACL) is the average value of the DoA assessment score determined by five experienced anaesthesiologists (i.e., attending physicians) based on clinical recordings and their own experience [27]. An experienced anaesthesiologist trained for many years with rich clinical experience can be familiar with health risks evaluation and accurately assess the DoA through clinical signs, surgical stimulations, the dose of the anaesthetic agent, etc. combined with his or her own clinical experience. Thus, such an expert can perform anaesthesia-related operations proficiently and correctly handle various problems in anaesthesia even if he or she is not in the operating room during surgery. The states of general anaesthesia are classified as anaesthesia induction, anaesthesia maintenance, and anaesthesia recovery, which refer to the gradual increase, stability, and gradual decrease of the anaesthesia depth, respectively. The obtained EACL value is a single number from 0 to 100, similar to the BIS (with 100 denoting ‘fully awake’ and 0 denoting ‘isoelectricity’). During surgery, the clinical information recorded included: (1) vital signs (e.g., HR, BP, SpO_2_), (2) anaesthetic events, including induction, LOC, intubation, maintenance, ROC and extubation of anaesthesia, addition of muscle relaxant drugs, and airway management, (3) surgical events, including the start and end of the surgical procedure and the occurrence of noxious stimulus, (4) other clinical signs, including unusual responses, movement, and arousability under induction and recovery, and (5) any other related events, such as lacrimation, sweating, and patient demography.

### ECG preprocessing

Body movements and medical device frequency noise are the main artifacts in ECG recordings. These artifacts seriously affect the analysis results of the ECG signals. Therefore, data preprocessing is essential for distinguishing different anaesthesia states and can normalize and facilitate subsequent analysis. The specific process is detailed in additional file 1(1).

### Frequency-domain algorithm

Wavelet transform is a typical nonlinear analysis technique and one of the most useful methods for biological signal analysis, especially for continuous signals with various frequency features [37]. Therefore, in this study, discrete wavelet transform was used for the frequency domain analysis of the HRV power. The calculation formula for the HRV power is detailed in additional file 1(2). Entropy, as a nonlinear dynamic parameter measuring the incidence of new information in a time series, can be described as a regularity or degree of randomness indicator. SampEn is an improved algorithm based on approximate entropy. The calculation formula for the SampEn is detailed in additional file 1(3).

### Machine learning algorithms

**Fig. 1:**
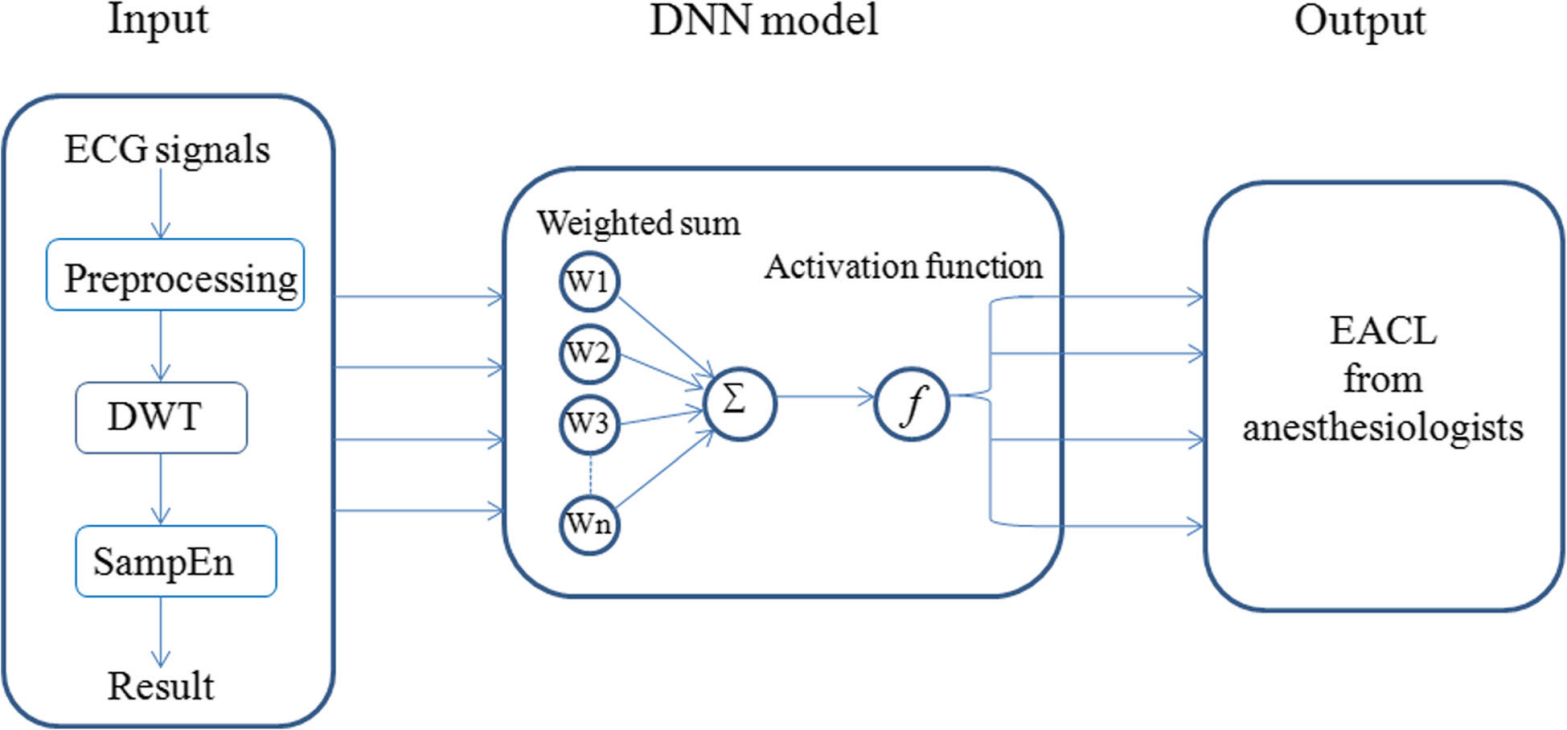
Flowchart depicting the proposed deep neural network model. DWT: discrete wavelet transform; DNN: deep neural network; EACL: expert assessment of consciousness level

Logistic regression is a classification algorithm used to predict the probability of classifying dependent variables. A support vector machine is a supervised learning algorithm that can be applied to classification problems. The calculation formula for the support vector machine approach is detailed in additional file 1(4). A decision tree is a multi-classification supervised learning algorithm. The calculation formula for the decision tree method is detailed in additional file 1(5). An artificial neural network is a nonparametric parallel computing model, which is similar to the neural structure of the human brain [38]. It usually consists of an input layer, a hidden layer, an output layer, and numerous interconnected nodes in multiple layers. The deep neural network developed from the artificial neural network was used in this study. The flowchart of the deep neural network construction is shown in Fig. 1. The deep neural network is detailed in additional file 1(6).

### Performance analysis

The performance of four models was quantified based on the results of cross-validation using the precision, recall, and classification accuracy. Precision is defined as the ratio of the number of correct classifications of an anaesthesia state to the total number of classifications of the same type of anaesthesia state. Recall is defined as the ratio of the number of correct classifications of an anaesthesia state to the number of actual occurrences of this anaesthesia state. Classification accuracy is defined as the ratio of the total number of correctly identified anaesthesia states to the sum of all anaesthesia states. The calculation formulas for the precision, recall, and classification accuracy are detailed in additional file 1(7).

### Statistical analysis

There are no standardized methods for sample size calculation based on machine learning algorithms. Thus, the sample size calculations in this pilot feasibility study were based on previous reports [32, 34]. Herein, the sample size was 23 cases, corresponding to a total of 46,000 datasets with an average of 2000 datasets per patient. 80% of the datasets, i.e., 36,800 datasets, were used to train the model. 20% of the datasets, i.e., 9200 datasets, were used to test the model. Statistical analyses were performed using SPSS 22.0 (SPSS Inc., Chicago, IL) and Python (version 3.6.5) software. Data were expressed as mean (SD) or percentage, where appropriate. Ternary classification outcome parameters were expressed as events (percentages). The data are presented in the form of tables, box-and-whisker diagrams, and correlation graphs. In addition, we calculated the distribution of the four features in the three anaesthesia states. The Pearson’s correlation coefficient between the EACL and the four features of the deep neural network model was also calculated to estimate the efficacy of the proposed method. The performances of four classification methods were compared: the logistic regression, support vector machine, decision tree, and proposed deep neural network methods. Owing to the small sample size in this study, the sample does not satisfy a normal distribution. Therefore, the four classification methods were compared using the Chi-square test. *p* < 0.05 was considered statistically significant.

## Results

### Primary outcome

**Fig. 2:**
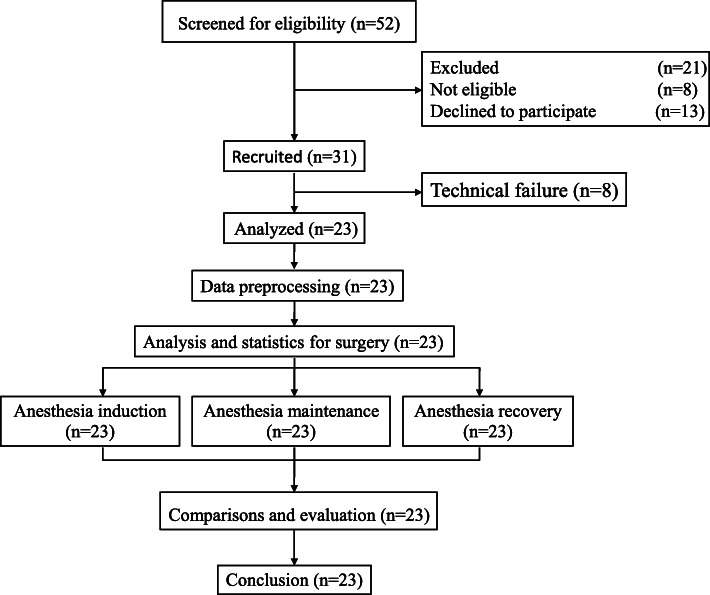
Study protocol

The clinical data of twenty-three adult patients were analysed in this study. The details of the selection procedure are shown in Fig. 2. Patient demographics and clinical characteristics are shown in Table 1. LOC was determined as no response to the command ‘name, name, open your eyes’. When LOC appeared during anaesthesia induction, the anaesthesiologist marked ‘LOC’ on the anaesthetic recording sheets immediately. The years of experience of five experienced anaesthesiologists are shown in Table 2. The deep neural network structure used in this study consisted of four layers: an input layer with four nodes, a hidden layer with ten nodes, a second hidden layer with seventeen nodes, and an output layer with one node. There were no cases of intraoperative awareness in this study.

**Table 2:** The years of experience of five experienced anaesthesiologists

	Anaesthesiologist A	Anaesthesiologist B	Anaesthesiologist C	Anaesthesiologist D	Anaesthesiologist E
years of experience	11	15	16	16	20

The precision and recall values of the anaesthesia induction, maintenance, and recovery states of the datasets for 23 cases are listed in Table 3. In addition, the classification accuracies of the three different anaesthesia states were obtained through the calculation of the recall and precision. The deep neural network model yielded a classification accuracy of 90.1%, whereas the logistic regression, support vector machine, and decision tree approaches yielded classification accuracies of 86.2, 87.5, and 87.2%, respectively. The accuracy of the deep neural network was higher than those of the logistic regression (*p* < 0.05), support vector machine (*p* < 0.05), and decision tree (*p* < 0.05) approaches. A comparison of the logistic regression, support vector machine, decision tree, and deep neural network methods is presented in Table 3. In addition, the precision and recall of the four models during the anaesthesia induction and recovery states were lower than those during the maintenance state.

**Table 3:** Comparison of logistic regression, support vector machine, decision tree, and deep neural network

	Precision of anaesthesia induction	Recall of anaesthesia induction	Precision of anaesthesia maintenance	Recall of anaesthesia maintenance	Precision of anaesthesia recovery	Recall of anaesthesia recovery	Classification accuracy
LR	55.1%	81.2%	94.6%	94.1%	46.3%	47.5%	86.2%
SVM	55.7%	80.1%	95.1%	94.6%	47.1%	46.8%	87.5%
DT	56.1%	80.9%	95.6%	94.8%	47.3%	47.1%	87.2%
DNN	58.1%	88.1%	96.0%	94.7%	56.6%	57.8%	90.1%

*LR* logistic regression. *SVM* support vector machine. *DT* decision tree. *DNN* deep neural network

### Secondary outcomes

**Fig. 3:**
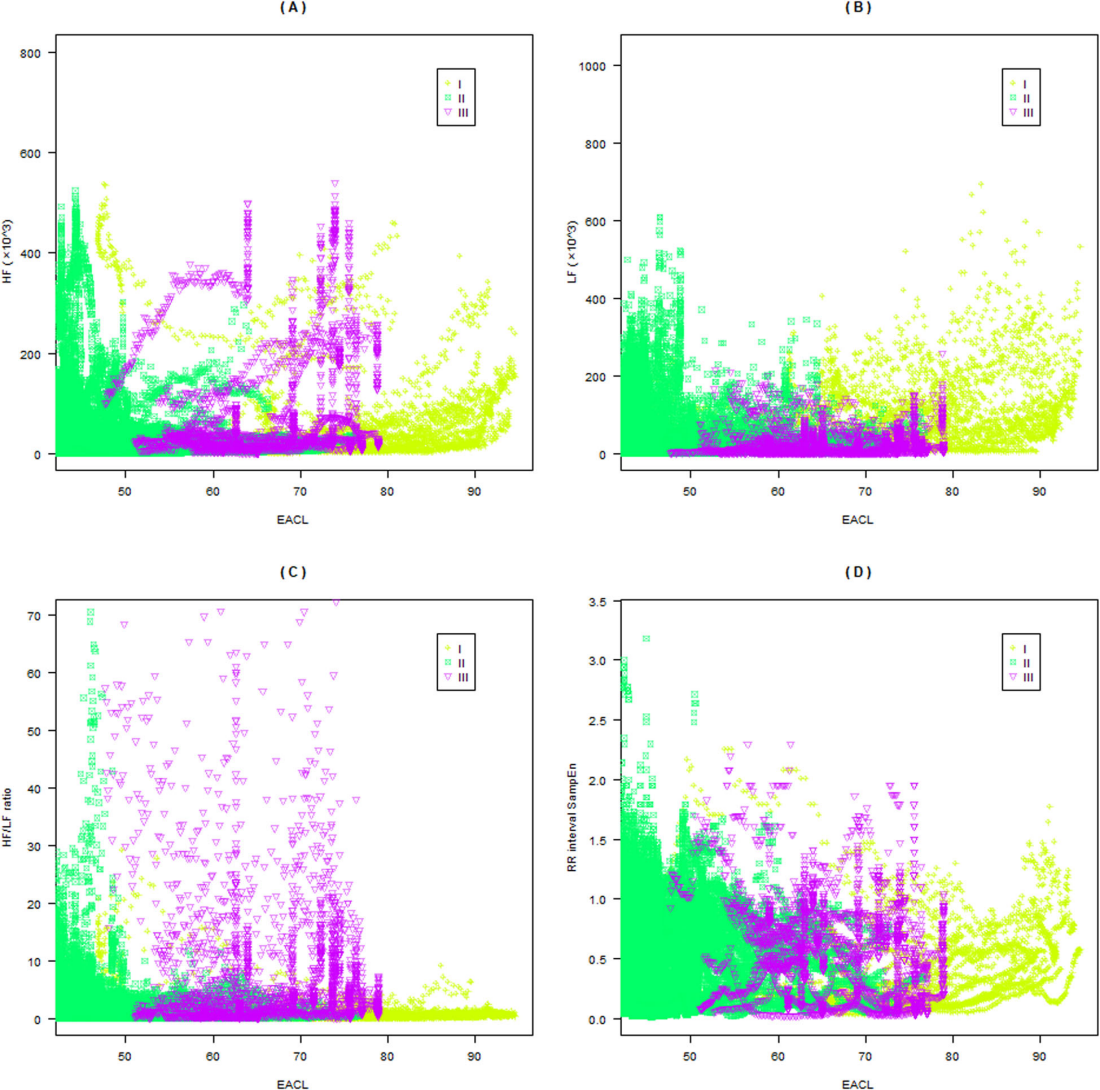
Correlations between the four features and EACL. **a**^_^**d** Correlations of HF, LF, ratio of HF/LF, and RR interval SampEn with the EACL, respectively. I, II, and III represent anaesthesia induction, anaesthesia maintenance, and anaesthesia recovery, respectively. EACL: expert assessment of consciousness level; HF: high-frequency; LF: low-frequency; HF/LF: high-to-low-frequency ratio

**Fig. 4:**
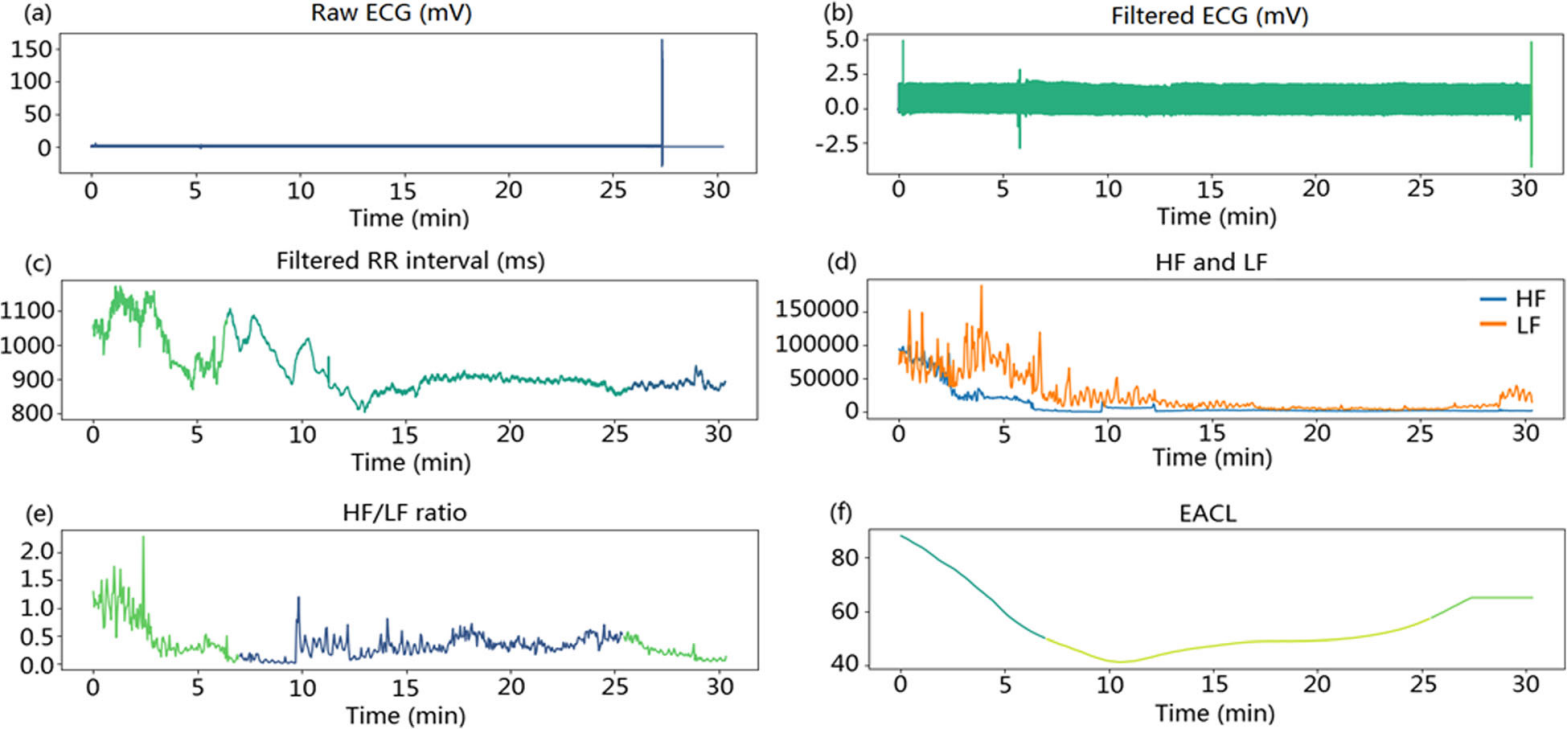
ECG data for the proposed method. **a**–**c** Raw ECG with visible artifacts, filtered ECG with tiny artifacts, and filtered RR intervals. **d**–**e** HF, LF, and ratio of HF/LF. **f** EACL within the sampling period. HF: high-frequency; LF: low-frequency; HF/LF: high-to-low-frequency ratio; EACL: expert assessment of consciousness level

In this study, four features of the HRV were selected as the input of the deep neural network model. Specifically, these were the HF, LF, HF/LF ratio, and SampEn of the RR interval. The EACL was used as the reference output. Figure 3 shows a clear correlation between the HF, LF, HF/LF, RR interval SampEn, and EACL. There are positive correlations between the HF (r = 0.221, *p* < 0.05), LF (r = 0.238, *p* < 0.05), and HF/LF (r = 0.106, *p* < 0.05) and the EACL. There is a negative correlation between the RR interval SampEn and the EACL (r = − 0.053, *p* < 0.05). Therefore, these features can be used for the construction of the deep neural network model. Interestingly, the four features are mainly distributed in the EACL value range of 40^_^80. In addition, Fig. 4 shows the original ECG signal, filtered ECG signal, filtered RR interval, HF, LF, HF/LF ratio, and EACL in the same time period. The voltage of the filtered ECG signal mainly varied between 0 and 2.5 mV. During the sampling period, the voltage of the ECG was relatively stable. The filtered RR interval, HF, LF, and HF/LF ratio were significantly reduced before reaching a relatively stable level. The trend of change in the three frequency features was similar to that of the EACL.

### Exploratory outcomes

**Fig. 5:**
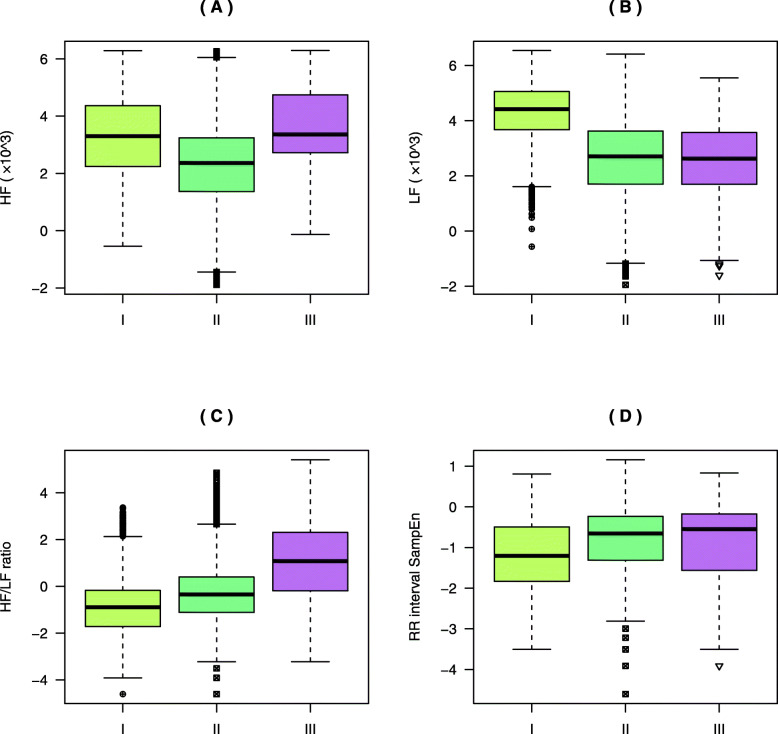
Comparison between anaesthesia states. The Y-axis is logarithmically transformed. (A)–(D) Distributions of (**a**) HF, (**b**) LF, (**c**) the ratio of HF/LF, and (**d**) the RR interval SampEn values. I, II, and III represent anaesthesia induction, anaesthesia maintenance, and anaesthesia recovery, respectively. Vertical coordinates represent the four feature values. HF: high-frequency; LF: low-frequency; HF/LF: high-to-low-frequency ratio; EACL: expert assessment of consciousness level

Figure 5 depicts the distribution characteristics of the four features under three different anaesthesia states. The HF during the anaesthesia induction state is significantly higher than that of the anaesthesia maintenance state (*p* < 0.001). The HF during the recovery state is significantly higher than those of the anaesthesia maintenance (*p* < 0.001) and anaesthesia induction states (*p* < 0.001). Moreover, the LF gradually decreases during the three anaesthesia states. The HF/LF ratio during the anaesthesia recovery state is significantly higher than those of the anaesthesia induction and maintenance states (*p* < 0.001). Finally, the SampEn of the RR interval gradually increases under the three anaesthesia states.

## Discussion

This study proposed a novel method for distinguishing different anaesthesia states based on four HRV-derived features in the time and frequency domains, combined with a deep neural network. In addition, this study compared the proposed deep neural network model with logistic regression, support vector machine, and decision tree in terms of the accurate classification of three anaesthesia states. The datasets of 23 patients who underwent general anaesthesia were used for assessing the proposed method. We used the precision, recall, and accuracy for model performance assessment. Each of the four models provided high accuracy in classifying the three anaesthesia states. However, the accuracy of the proposed method outperformed the three conventional methods. This suggests that, by testing the datasets obtained from multiple HRV-derived features, it is possible to reliably predict the anaesthesia states based on machine learning algorithms.

Most research has assessed the DoA based on EEG features and machine learning algorithms; however, few studies have distinguished different anaesthesia states using HRV-derived features based on machine learning algorithms. Several studies were developed to predict the DoA using combinations of multiple EEG features and logistic regression [31], support vector machine [32], decision tree [33], and artificial neural network [34]. We adopted a multidimensional approach using logistic regression, support vector machine, decision tree, and deep neural network methods and four HRV-derived features to distinguish different anaesthesia states. One of the major findings in this study is that, like EEG features, HRV-derived features based on machine learning algorithms can also distinguish different anaesthesia states. Moreover, Liu et al. used only the similarity and distribution index of HRV based on an artificial neural network to assess the DoA [21]. The similarity index of HRV can distinguish between the waking and isoflurane anaesthesia states [39]. Our findings are consistent with these results in that HRV-derived features selected can also be used to distinguish different anaesthesia states. However, our study differs from previous ones as we used multiple HRV-derived features and machine learning algorithms.

To assess the accuracy of these machine learning algorithms, we selected the EACL as the evaluation criterion for distinguishing different anaesthesia states. The EACL adopted in this study is a method of clinical evaluation performed by five experienced anaesthesiologists for evaluating the DoA. As current DoA monitors such as the BIS are based on probabilistic approaches, clinical assessment of the level of consciousness remains the golden standard [40]. In addition, current DoA monitors based on EEG features have accuracy limitations [11, 13–15]. To improve the accuracy of DoA estimation, previous studies used the EACL as the evaluation criterion for DoA assessment. Liu et al. employed the EACL as the reference standard for the output of an artificial neural network to assess the DoA [21]. Meanwhile, Jiang et al. used SampEn analysis of EEG signals based on an artificial neural network and EACL to model patient consciousness levels [27]. However, our study shows that HRV-derived features based on a deep neural network and EACL can be used to distinguish different anaesthesia states. Thus, in addition to current DoA monitors, the EACL is also a reliable method of identifying anaesthesia states.

Our findings show a clear correlation between the four HRV-derived features and EACL. These HRV-derived features are also closely related to different anaesthesia states. As the HRV is controlled by the central nervous system, the DoA should be considered to assess the effects of anaesthetics on HRV [20]. To date, it is considered that the LF reflects the parasympathetic and sympathetic systems, whereas the HF and entropy are mediated primarily by the parasympathetic system [41, 42]. In addition, some previous studies have shown that HRV-derived features, including the entropy, HF, LF, and HF/LF, could reflect changes in the DoA. Propofol decreases the entropy and HF in a BIS-dependent manner [20], and it is related to the relative decrease in the HF, increase in the LF, and significant decrease in HF/LF during the anaesthesia induction state [43]. However, abrupt increases in the LF and HF are related to moment patients become responsive to verbal commands during the anaesthesia recovery state [44], whereas our study shows that the HF increased and LF decreased. In addition, the results in this study indicate that the changes in the four HRV-derived features could reflect the change of anaesthesia states. Therefore, these HRV-derived features are reliable features of distinguishing anaesthesia states. However, the correlation between a single feature and the EACL was not strong, and the synergy between the four features can be improved to classify the different anaesthesia states. Thus, to implement the proposed method in clinical settings, different features need to be selected for subsequent research and the accuracy of the prediction method must be improved.

The optimal DoA prediction method should have high accuracy and should not be influenced by interference from irrelevant signals. Our findings show that, with the help of multiple HRV-derived features and machine learning algorithms, distinguishing different anaesthesia states is feasible. In addition, the proposed method has several advantages. First, ECG signals are more stable and less susceptible to noise than EEG signals. Further, the electrode sensors used for ECG signal acquisition are cheaper than those for EEG signal acquisition, rendering ECG a more cost-effective method. More importantly, our method may be a useful adjunct in monitoring DoA based on EEG features and is expected to assist anaesthesiologists in the accurate evaluation of the DoA.

Although promising, there are several limitations and a need for further improvement. First, we did not distinguish nociceptive effects and other physiological parameters, such as hemodynamic and respiratory variables, on HRV. However, our findings provide important references to guide future investigations. Second, we only explored four HRV-derived features as the inputs of the deep neural network in this feasibility study. We limited these features as they contain both time- and frequency-domain characteristics of HRV. In addition, cross-validation was used to train and test the model to avoid over-fitting, ensure model generalization, and improve the performance of the deep neural network. Additionally, we considered the impact of inter-clinician variability on the performance of the deep neural network model. To minimize personal error, the mean values of the DoA assessment score determined by five experienced anaesthesiologists were used as the reference standard for the output of the deep neural network. Third, the DoA in this study was classified into the three anaesthesia states in the deep neural network model. It is necessary to explore new methods of DoA evaluation with higher precision, better performance, and more classifications (e.g., four or more states) in subsequent work. Fourth, the number of patients used in this study was limited. Increasing the number of patients could improve the performance of our proposed method. Besides, owing to the emergence of agitation during the recovery period, the electrodes on the chest walls of eight patients fell off, and the ECG data collection was interrupted, causing technical failure.

## Conclusions

In conclusion, this study combined multiple HRV-derived features, including three frequency-domain features and one time-domain feature, with four machine learning algorithms to identify the three anaesthesia states. The proposed method could accurately distinguish between different anaesthesia states and outperformed three traditional machine learning algorithms. Our method provides a useful reference for supplementing DoA assessment based on EEG features and is expected to assist anaesthesiologists in the accurate evaluation of the DoA. Other physiological signals, such as EEG, could be incorporated into the proposed method to further improve the accuracy of DoA estimation.

## Supplementary Information

**Additional file 1.** Supplementary information on methods.

## Data Availability

The datasets are not publicly available, but available from the corresponding author on reasonable request.

## Abbreviations

DoA: Depth of anaesthesia
EEG: Electroencephalogram
HRV: Heart rate variability
DWT: Discrete wavelet transform
DNN: Deep neural network
HF: High-frequency power
LF: Low-frequency power
HF/LF: High-to-low-frequency power ratio
RR interval: the interval between R peaks in two adjacent heartbeats of the ECG
LOC: Loss of consciousness
ROC: Recovery of consciousness
EACL: Expert assessment of consciousness level
BIS: Bispectral index
ECG: Electrocardiogram
HR: Heart rate
BP: Blood pressure
SpO_2_: Peripheral oxygen saturation
SampEn: Sample entropy
ASA: American Society of Anaesthesiology
Hz: Hertz
SD: Standard Deviation
BMI: Body mass index
