# Supplementary material for: Heart rate variability-derived features based on deep neural network for distinguishing different anaesthesia states
Source: BMC Anesthesiol. 2021 Mar 2;21:66. doi: 10.1186/s12871-021-01285-x (PMC7923817; doi:10.1186/s12871-021-01285-x)
Supplement: Supplementary file 1 — Additional file 1. Supplementary information on methods. [file 12871_2021_1285_MOESM1_ESM.docx]

**(1) ECG preprocessing**

Preprocessing usually includes data format conversion, noise cancellation, and data rearrangement. In this study, the outliers beyond the threshold (85 ms) were first removed by comparing the current RR interval, the interval between R peaks in two adjacent heartbeats of the ECG, in the ECG signal with the average of the previous ten sampling points. Second, a low-pass (16 Hz) filter and a high-pass (8 Hz) filter were used consecutively to remove the baseline drift and frequency noise from the medical devices. The filters do not interfere in the frequency information of the ECG signals. The frequency information (3-45 Hz) was retained for resampling. Third, the average method was used to remove the electromyogram (EMG) artifacts and transient high-amplitude artifacts [1]. The average method compares whether the average value of the current RR interval and the past 10 sampling points is less than the threshold and removes the point if it exceeds the threshold. Fourth, the ECG data was resampled to 1.67 Hz [2]. In addition, some sampling points were far beyond the normal biological voltage, meaning only the sampling points from -5 to 5 mV were retained. Finally, 153-s epochs including 256 RR intervals were extracted from the artifact-free ECG. In this study, four kinds of features were used as the inputs of the deep neural network to evaluate the DoA. These include three frequency features and one SampEn feature. The HF, LF, and HF/LF ratio were calculated as the frequency domain features and SampEn of the RR interval as the entropy feature. In addition, four algorithms were applied to the ECG data processing.

**(2) Frequency-domain algorithm**

Wavelet transform is usually categorized into continuous wavelet transform and discrete wavelet transform. Due to the discontinuity of the RR interval sampling, discrete wavelet transform is used for time-frequency analysis. In addition, discrete wavelet transform is an effective wavelet transform analysis method and can be used for assessing DoA [3]. First, RR interval detection was performed according to Hamilton's method [4]. Second, 256 consecutive RR intervals were set as the time window of the discrete wavelet transform, with a translation step of 5 RR intervals. Third, the db4, a discrete wavelet of the Daubechies wavelets was set as the mother wavelet. The length of the db4 wavelet was set to eight and the vanishing moment to four. The wavelet decomposition level was set to seven. In addition, discrete wavelet transform was used for the time frequency analysis in this study, following the work of Shensa et al. [5]. The LF and HF were 0.04^_^0.15 Hz and 0.15^_^0.4 Hz, respectively. The frequency-domain features of interest were the HF, LF, and HF/LF ratio. The absolute values of the original HF and LF were retained in the frequency domain feature to classify different anaesthesia states.

The HRV power was defined as the sum of squares of the time domain coefficients at a certain frequency after the discrete wavelet transform. The calculation formula for the HRV power is as follows:

| $E_{s}=\left\langle x\left( n \right),x(n) \right\rangle=\sum_{n=-\infty}^{\infty} \left\vert x(n) \right\vert^{2}$. | (1) |
| --- | --- |

The discrete time signal $x(n)$ is defined as:

| $x\left( n \right)=\left\{ x_{n} \right\}$, | (2) |
| --- | --- |

where $x\left( n \right)$ is the *n*^th^ digit in the sequence $x$*,* and$n$ is an integer.

**(3) Sample entropy**

SampEn is an improved algorithm proposed by Richman and Moorman [6] based on approximate entropy, which reduces the deviation caused by self-matching. The SampEn function is a negative logarithm, indicating whether two similar sequences of m consecutive data points remain similar at the next point ($m+1$).

| $S\text{E}\left( m,r,N \right)=-\ln\left( \frac{C_{m+1}\left( r \right)}{C_{m}\left( r \right)} \right)$, | (3) |
| --- | --- |

The definition of $C_{m}$[7] is as follows:

| $C_{m}\left( r \right)=\frac{\left\{ number of all probable pairs\left( i,j \right)\mathrm{with}\mid x_{i}^{m}-x_{j}^{m}\mid<r,i\neq j \right\}}{\left\{ number of all probable pairs,i.e(N-m+1)(N-m) \right\}}$, | (4) |
| --- | --- |

where $\mid x_{i}^{m}-x_{j}^{m}\mid$ is the distance between points $x_{i}^{m}$ and $x_{j}^{m}$ in the dimension space, *m* and *r* denote the tolerable standard deviation of the time series, and *N* represents the length of the time series. Thus, the SampEn of the RR interval was calculated, and the analysis window was found to be consistent with that obtained using frequency domain analysis. In addition, 256 consecutive RR intervals were used as the time-domain window. We set the parameter to *m*= 5, and *r* is 0.3 times the standard deviation of the original signal in the current window.

**(4) Support vector machine**

Support vector machine is a linear classifier with the largest geometric interval in the feature space. In this study, we deployed classification modelling using the Lagrange multiplier method, while the specific calculation steps follow those in previous research [8].

**(5) Decision tree**

Decision tree can be used not only for classification problems but also regression problems, and is represented by a tree structure in which each internal node represents a judgment condition of an attribute, each branch represents the output of the judgment result, and each leaf node represents a classification result. The construction of a decision tree aims to classify the conditions by judging the size of the information gain under a certain feature. The formula of the information gain is determined by the difference of the entropy minus the conditional entropy. The formula of the conditional entropy is as follows:

| $H\left( Y \vert X \right)=\sum_{i=1}^{n} p_{i}H\left( Y \vert X=x_{i} \right)$. | (5) |
| --- | --- |

Conditional entropy represents the uncertainty of the random variable $Y$ under the condition that the random variable $X$ is known.

Therefore, the formula of the information gain is as follows:

| $g\left( Y,X \right)=H\left( Y \right)-H\left( Y \vert X \right)$, | (6) |
| --- | --- |

**(6) Deep neural network**

The nodes of an artificial neural network in the input layer receive external information, whereas the output layer outputs the result. Between the input and output layers, there are usually one or more hidden layers used to identify complex features in the data [9]. Thus, artificial neural networks support continuous self-learning and error correction, and they can analyse new problems and obtain optimal results. Supervised and unsupervised learning are the two learning rules in artificial neural networks.

Owing to the use of four HRV-derived features as the input features of the model, the input layer includes four neural nodes. The output layer only includes one neural node. Two hidden layers and neural nodes are obtained by repeatedly adjusting the parameters, model training, and testing simultaneously. The process of model optimization included dividing the ECG datasets into training and test sets according to a ratio of 8:2, five-fold cross-validation, dropout adjustment (random shielding of 20% of the neural nodes during training), and epoch adjustment (from 100 to 300). The accuracy of two hidden layers is higher than that of a single hidden layer. However, adding another hidden layer does not improve the accuracy.

There are ten neural nodes in the first hidden layer and seventeen neural nodes in the second. Furthermore, the back-propagation algorithm, which is the most commonly used learning algorithm [8], was implemented in the proposed deep neural network model. For the construction of the deep neural network model, the four features of the HRV were set as the input, and the EACL was used as the reference standard for the output.

**(7) Performance analysis**

The 46000 datasets of 23 cases in this study were divided into training and test datasets. 80% of the datasets was used to train the model, and 20% of the datasets was used to test the model. Training and testing were performed simultaneously to reduce model over-fitting. Owing to the limited number of samples, a 5-fold cross-validation strategy was used to evaluate the generalization ability of the predictors.

| $P_{i}=\frac{N_{i,detceted}}{N_{i,total}}$, | (7) |
| --- | --- |

$N_{i,detceted}$ represents the number of correct classifications of an anaesthesia state;$N_{i,total}$ represents the total number of classifications of the same type of anaesthesia state. Where *i* represents the three anaesthesia states (anaesthesia induction, anaesthesia maintenance, anaesthesia recovery).

| $R_{i}=\frac{N_{i,detceted}}{N_{m,total}}$, | (8) |
| --- | --- |

$N_{m,total}$ represents the number of actual occurrences of this anaesthesia state.

Where *m* is the actual number of one anaesthesia state.

| $ACC=\frac{N_{detceted}}{N_{total}}$. | (9) |
| --- | --- |

$N_{detceted}$represents the total number of correctly identified anaesthesia states; $N_{total}$represents the sum of all anaesthesia states.

**References**

1. Fraser GD, Chan ADC, Green JR, Macisaac D. Removal of electrocardiogram artifacts in surface electromyography using a moving average method. IEEE International Symposium on Medical Measurements & Applications. 2012:1-4.

2. Berger RD, Akselrod S, Gordon D, Cohen RJ. An efficient algorithm for spectral analysis of heart rate variability. IEEE Trans Biomed Eng. 1986;33(9):900-4.

3. Tai NK, Peng W, Yan L. Monitoring the Depth of Anesthesia Using Discrete Wavelet Transform and Power Spectral Density. Rough Sets and Knowledge Technology. 2009:350-357.

4. Hamilton P. Open source ECG analysis. Computers in Cardiology. 2002;29:101−4.

5. Shensa MJ. The discrete wavelet transform: wedding the a trous and Mallat algorithms. Ieee T Signal Proces. 1992; 40(10):2464-82.

6. Richman JS, Moorman JR. Physiological time-series analysis using approximate entropy and sample entropy. Am J Physiol Heart Circ Physiol. 2000;278(6):H2039-49.

7. Jiang GJ, Fan SZ, Abbod MF, Huang HH, Lan JY, Tsai FF, et al. Sample entropy analysis of EEG signals via artificial neural networks to model patients’ consciousness level based on anesthesiologists experience. BioMed Res Int. 2015;2015:343478.

8. Gu Y, Liang Z, Hagihira S. Use of multiple EEG features and artificial neural network to monitor the depth of Anesthesia. Sensors (Basel) 2019; 19(11):2499.

9. Zhang G, Hu MY, Patuwo BE, Indro DC. Artificial Neural Networks in Bankruptcy Prediction: General Framework and Cross-Validation Analysis. Eur J Oper Res.1999;116(1):16-32.
